# A Jejunal Dieulafoy Lesion: Rare Case Necessitating Surgical Intervention

**DOI:** 10.1016/j.ijscr.2020.06.098

**Published:** 2020-06-25

**Authors:** Misbah Yehya, Oksana Mayovska, Amanda Flick, Zbigniew Moszczynski

**Affiliations:** Department of Surgery, Carepoint Health Bayonne Medical Center, Bayonne, NJ, USA

**Keywords:** Dieulafoy lesion, Gastrointestinal bleeding, Hemorrhagic shock, Hematochezia, Jejunum

## Abstract

**Introduction:**

Dieulafoy lesions are enlarged atypical submucosal vessels that erode the superimposing epithelium in the absence of a primary ulcer. They occur predominantly in the gastric mucosa; however, cases have been seen throughout the gastrointestinal (GI) tract, and rarely, the jejunum. These lesions can cause massive GI hemorrhage leading to shock.

**Case Presentation:**

We present a healthy 19-year-old male who arrived at our institution’s emergency department with multiple episodes of hematemesis and hematochezia that began earlier that morning. The patient was resuscitated and underwent a computerized tomographic (CT) angiography that did not identify any areas of active extravasation. The patient was then taken for an emergent upper and lower endoscopy that was inconclusive. He was subsequently sent for a tagged red blood cell scan, which demonstrated active bleeding in the proximal jejunum. Shortly thereafter, the patient began to decompensate requiring additional blood products and vasopressors. The decision was made for immediate operative intervention, which identified the bleeding Dieulafoy lesion (confirmed by histopathology) in the jejunum.

**Discussion:**

Dieulafoy lesions are rare with an initial presentation of upper or lower GI bleeding, generally treated with endoscopic intervention. They are predominately found in the stomach or duodenum. When no clear source is identified by endoscopy, further diagnostic testing may be of value. Various imaging modalities exist; however, CT angiography or radionuclide scanning are particularly sensitive and can be beneficial in localizing the bleed when preparing for operative intervention.

**Conclusion:**

With advances in endoscopic techniques, surgical intervention is rarely performed for a Dieulafoy lesion. If endoscopy is unsuccessful, additional imaging can be obtained to localize the source of bleeding. However, in emergent cases, when additional imaging cannot be obtained due to lack of time or resources, adequate resection of the lesion should be performed for complete resolution of the disease process. Based on the case presentation and pathologic findings, this case provides further insight into Dieulafoy lesions and the rare need for surgical management.

## Highlights

• Dieulafoy lesions are aberrant submucosal lesions that, in rare circumstances, can result in massive gastrointestinal bleeding.
• These lesions are particularly rare in the jejunum, thus usual diagnostic and therapeutic modalities such as endoscopy may prove difficult.
• Additional imaging such as angiography or radionuclide scanning can be beneficial in identifying the source of bleeding.
• If advanced endoscopic instruments are unavailable at one’s facility, the surgeon must consider operative intervention for life-saving measures.
• The operative findings and management discussed can guide clinicians unexposed to this disease process when surgical intervention is imminent.

## 1 Introduction

The first reports of a Dieulafoy lesion in the late 1800s were in concordance with substantial gastric hemorrhage secondary to a gastric vascular malformation. These lesions have since been reported in other areas of the gastrointestinal tract; however, they remain particularly rare in the small bowel, with only 1% of case reports documenting lesions in the jejunum.

A Dieulafoy lesion is a dilated abnormal submucosal artery that erodes the overlying epithelium in the absence of a primary ulcer [1]. These lesions do not follow the typical pattern of mucosal capillaries thereby leading to much larger caliber vessels making them prone to injury and bleeding. The most common location reported is the stomach, along the lesser curvature, although literature review has also confirmed lesions in the esophagus, duodenum, and rarely, the jejunum.

The etiology remains unknown however exacerbating factors leading to gastrointestinal bleeding have been hypothesized which include non-steroidal anti-inflammatory drug (NSAID) usage and alcohol abuse [1]. The initial diagnostic modality is by endoscopic identification via esophagogastroduodenoscopy (EGD). They can usually be identified in the acute setting as an actively bleeding, pulsatile lesion without visible ulceration. In the absence of active bleeding, a Dieulafoy lesion may appear as a raised nipple or visible vessel without an associated ulcer; however, the aberrant vessel may not be visible to the naked eye unless there is active bleeding from the site [2].

Treatment can range from endoscopic hemoclipping, cautery, or epinephrine injections to control the bleeding, but this is limited to the length of the endoscope and not ideal for patients with lesions beyond the duodenum. Push single and double balloon enteroscopy can be attempted with lesions beyond the duodenum; however, its limitations include the institutional availability of said endoscope and the endoscopist’s technical skills. Furthermore, these lesions can rebleed leading to multiple endoscopic attempts at hemorrhagic control. Surgical intervention, especially in the setting of shock, recurrence with multiple endoscopic procedures, or no clear source of bleeding, is the most appropriate and ideal management and can lead to control of bleeding, patient stabilization, and complete resolution of symptomatology.

## 2 Case report

Our patient is a 19-year-old male with no significant past medical or surgical history who presented to the emergency department by ambulance after a sudden onset of bright red bleeding per rectum and hematemesis. The patient stated his symptoms began the morning of his admission and were associated with nausea, abdominal cramping, and dizziness. He denied any prior episodes of similar symptoms. Patient denied any history of drug or alcohol use or family history of hematological and gastrointestinal disorders such as Crohn’s disease.

His admission laboratory work-up revealed a hemoglobin of 10.0 and a white blood cell count of 12.3. Initial imaging including a computed tomography (CT) angiography of the abdomen and pelvis did not show any areas of active extravasation. While in the emergency department, the patient had two additional episodes of hematochezia and was transferred to the intensive care unit (ICU) for adequate resuscitation with crystalloids and blood products.

**Fig. 1:**
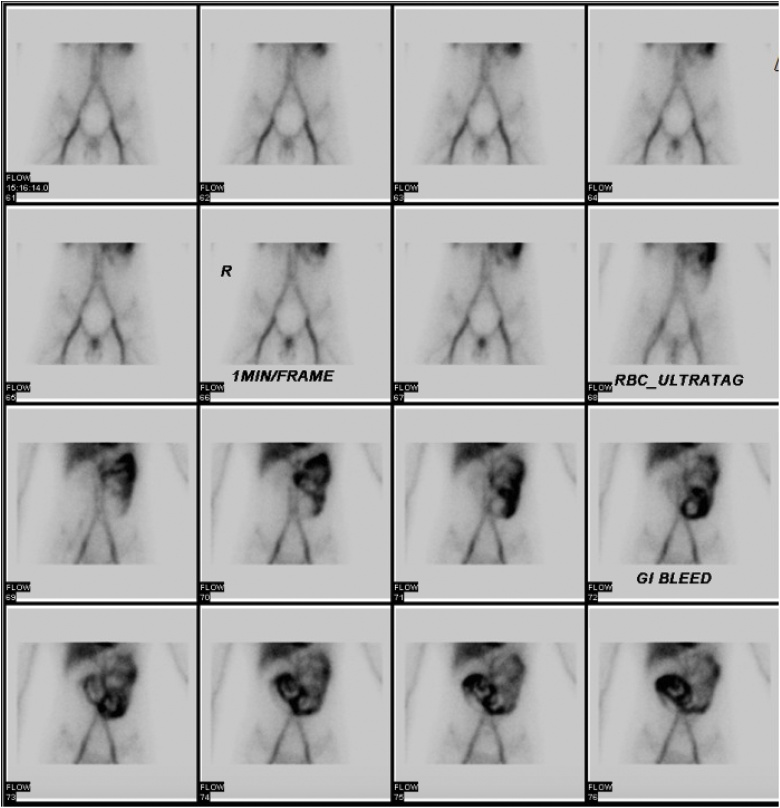
Tagged red blood cell scan showing tracer activity in the left upper quadrant raising suspicion for a proximal jejunal bleeding site.

The patient was consented for an emergent endoscopy/colonoscopy. The EGD was unremarkable; however, the colonoscopy showed a significant amount of bright red blood throughout the colon from rectum to terminal ileum without a clear source of bleeding. He was taken for a tagged red blood cell scan to identify the source while continuously being transfused with Packed Red Blood Cells (PRBC) and Fresh Frozen Plasma (FFP) to maintain hemodynamic stability. The resulting scan revealed abnormal tracer activity in the left upper quadrant with the proximal jejunum as the most likely source of bleed [Fig. 1].

At this time, he had additional episodes of hematochezia with a drop in his systolic blood pressure. His repeat hemoglobin was now 7.3 and he began requiring vasopressors. Because of the location of the GI bleed in the jejunum where vasculature is variable and with the initial angiography unable to detect any source of bleeding, the interventional radiologist was not certain embolization would be a reliable method of treatment for our patient.

**Fig. 2:**
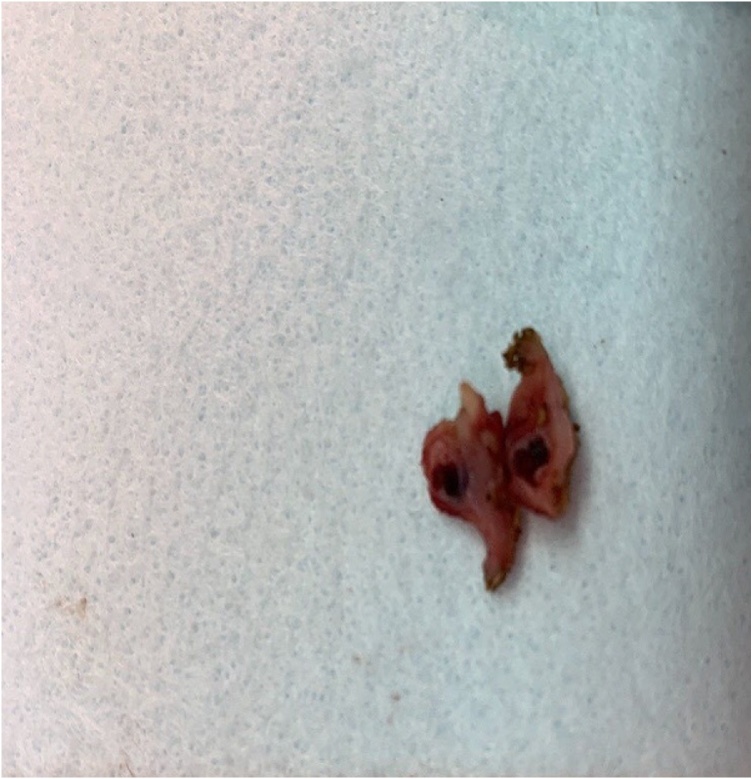
The gross cross-sectional image of the resected jejunal lesion showing a dilated segment of artery filled with blood.

With his hemodynamic instability progressing, the decision was made to take the patient emergently to the operating room where he underwent an exploratory laparotomy with the general surgeon and surgical residents. During this time, the small bowel was run from the terminal ileum to the ligament of Treitz, paying close attention to the jejunum. We were able to visualize intraluminal blood coursing from the proximal/mid- jejunum to terminal ileum. Proximal to this site, no intraluminal blood was noted. Additionally, a 1 cm palpable lesion was identified in the jejunum. An enterotomy at the proximal jejunum confirmed the presence of a small, nonbleeding, raised nipple-like lesion [Fig. 2]. A small bowel resection including the lesion with primary anastomosis was performed. Upon careful inspection, no additional lesions were identified. The patient tolerated the procedure and was taken back to the ICU for close hemodynamic monitoring.

**Fig. 3:**
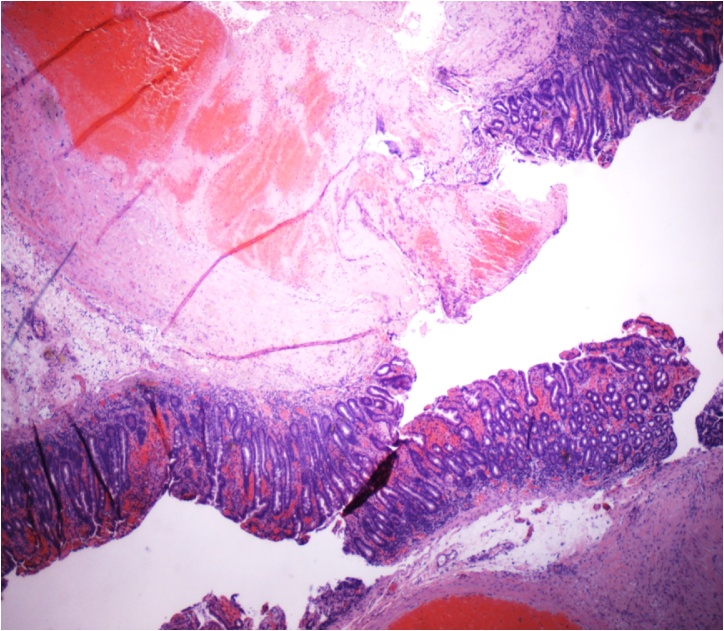
The histopathological slide of small bowel mucosal erosion with underlying dilated tortuous segments of artery filled with blood consistent with a Dieulafoy lesion.

The small bowel specimen was sent for pathology which revealed a small bowel mucosal erosion with underlying dilated tortuous segments of artery filled with blood consistent with a Dieulafoy lesion [Fig. 3].

On postoperative day 1, the patient was extubated. During his postoperative course he subsequently remained hemodynamically stable. He received a total of 8-units of PRBCs, 2-units of FFP, and 1-unit of platelets during his admission, primarily prior to the surgical intervention. The patient was stable for transfer out of the ICU on postoperative day 3. His diet was resumed and continually advanced as tolerated to a regular diet. On postoperative day 5, the patient was discharged home in stable condition. He was subsequently seen on follow up visit with complete resolution of his symptoms, fully healed surgical incision, and tolerating a regular diet. Our work has been reported in line with the SCARE criteria [13].

## 3 Discussion

Dieulafoy lesions are defined as active arterial bleeding or an adherent clot on an underlying vessel in the absence of an ulcer [3]. They are extremely rare, especially in the jejunum, with less than 50 reported cases in the United States. The etiology remains unknown but some studies suggest peptic ulcer disease, NSAID use, and/or alcohol abuse play a role in aggravating these lesions resulting in hemorrhage; however, in the younger population, no causal establishment has been documented [1].

These lesions are typically seen in the stomach and duodenum with treatment involving endoscopic intervention as the diagnostic modality of choice to obtain adequate control of the bleeding vessels. The Endoscopic criteria for Dieulafoy lesions include several parameters: 1) active arterial spurting or micropulsative streaming from a tiny mucosal defect or through the normal surrounding mucosa; 2) visualization of a protruding vessel with or without active bleeding within a tiny mucosal defect or through the normal surrounding mucosa; and/or 3) fresh, densely adherent clot(s) with a narrow point of attachment to a tiny mucosal defect or to normal appearing mucosa [11].The type of intervention varies with one report advocating for mechanical hemostasis (endoclipping and band ligation) over injection therapy alone with respect to effective initial hemostasis and rates of recurrent bleeding [4]. Another trial also concluded the effectiveness of endoclipping when compared to epinephrine injection especially with rates of rebleeding, which were observed to be higher in the epinephrine group [5]. Of note, reports of endoscopic band ligation causing perforations of the stomach have been reported and should be used with caution, especially when considering the thickness of the small bowel wall making it a less favorable option [6,7]. Additionally, these patients often require multiple endoscopic interventions as the lesions have a tendency to rebleed. Endoscopy has changed the way we approach upper and lower gastrointestinal hemorrhage however it has limitations.

When the source of bleeding cannot be identified by an upper endoscopy and colonoscopy, it is termed an obscure gastrointestinal bleed (OGIB) and among others, a Dieulafoy lesion should always be considered in the differential diagnosis. Table 1 summarizes the differentials a clinician should consider when an OGIB is encountered [8]. In a hemodynamically stable patient, further imaging can be obtained to localize the source of bleeding.

**Table 1:** Differential diagnoses for occult gastrointestinal bleeds.

In the acute setting of an OGIB, imaging modalities such as CT angiography or radionuclide studies can guide the clinician towards the specific site of bleeding. CT angiography, for example, is quick and easy to perform at most institutions with the ability to detect bleeding rates at 0.5 mL/min [9]. Radionuclide studies are the most sensitive technique for detecting active GI bleeding with the ability to detect bleeding rates at 0.1 mL/min [10] and avoids any radiation exposure. These studies, although not therapeutic, can be done relatively rapidly and are able to detect the most probable site of bleeding. If time and patient stability permits, we recommend attempting a CT angiography or radionuclide scan prior to any surgical intervention to potentially localize the source and guide the surgeon intraoperatively. In our case, the radionuclide scan was more sensitive compared to the CT angiography in its ability to detect the area of active GI bleed, which helped guide our management.

Therapeutic intervention of an OGIB in a hemodynamically stable patient can be pursued with the use of a push single or double balloon enteroscopy that allows the endoscopist to visualize the small bowel and apply therapeutic modalities as needed. Unfortunately, this is highly dependent on the endoscopist’s technical experience and whether such devices are available at the presenting institution. Our institution did not have the resources for a push enteroscopy. For patients warranting further intervention, surgical evaluation is an appropriate alternative, especially in the setting of uncontrollable bleeding and hemodynamic instability.

Our patient was initially stable to undergo a radionuclide scan that demonstrated a source in the proximal jejunum; however, as he became hemodynamically unstable and was not appropriately responding to blood products, he was taken emergently to the operating room for an exploratory laparotomy. At the time of intervention, we relied on the visual appearance of the small bowel as well as palpation of any abnormal lesions in and around the jejunum. The surgical attending noted intraluminal blood that was not present proximal to the suspected lesion which guided the placement of the enterotomy. This can help decrease the amount of enterotomies and length of small bowel resection. The lesion itself may or may not be actively bleeding as Dieulafoy lesions are known to bleed intermittently. When not bleeding, the mucosa is normal appearing and the only abnormality may be a small raised, firm lesion. These lesions can be easily missed and careful handling must be done when running the small bowel. The standard treatment once identified consists of a small bowel resection with primary anastomosis which has shown to be successful for cases requiring operative intervention [12].

## 4 Conclusion

With increasing expertise and advances in technology, gastrointestinal bleeding is now largely managed with endoscopic intervention. When no clear source can be identified after endoscopic evaluation, this raises suspicion for a Dieulafoy lesion in the differential diagnosis. We discussed the various imaging modalities that can be administered to help localize the source, specifically, CT angiography and radionuclide scanning, because of their increased sensitivity for recognizing actively bleeding vessels. This case demonstrates the importance of pre-operative stabilization and imaging studies to localize the potential source of bleeding. While endoscopic treatment has become the standard approach for Dielafoy lesions, one cannot disregard definitive surgical management, particularly in an unstable patient. We describe our intraoperative findings in detail as a means of guidance for surgeons who encounter obscure GI bleeding in a facility where advanced endoscopic capabilities are unavailable.

*Written informed consent was obtained from the patient for publication of this case report and accompanying images. A copy of the written consent is available for review by the Editor-in-Chief of this journal on request. No outside funding was received to produce this publication. There were no conflicts of interest encountered during production of this publication*.

## Declaration of Competing Interest

There are no conflicts of interest that influence the submitted work.

## Funding

There were no study sponsors involved in the submitted work.

## Ethical approval

This is not applicable to the submitted work.

## Consent

Written informed consent was obtained from the patient for publication of this case report.

## Author contribution

Study Concept/Design – Misbah Yehya, Zbigniew Moszczynski

Data Collection - Misbah Yehya, Zbigniew Moszczynski

Writing the Paper – Misbah Yehya, Oksana Mayovska, Amanda Flick

## Registration of research studies

This is not applicable to our submitted work.

## Guarantor

The Guarantor’s are Misbah Yehya, Oksana Mayovska, Amanda Flick, and Zbigniew Moszczynski.
